# Bioinformatics Analysis and Functional Prediction of Transmembrane Proteins in *Entamoeba histolytica*

**DOI:** 10.3390/genes9100499

**Published:** 2018-10-16

**Authors:** Tamanna Anwar, Gourinath Samudrala

**Affiliations:** School of Life Sciences, Jawaharlal Nehru University, New Delhi 110067, India

**Keywords:** membrane transport, transporters, transmembrane, channel, ion transporters, primary active transporters, secondary carriers, group translocaters, protein-protein interaction, therapeutic targets, amoebiasis

## Abstract

*Entamoeba histolytica* is an invasive, pathogenic parasite causing amoebiasis. Given that proteins involved in transmembrane (TM) transport are crucial for the adherence, invasion, and nutrition of the parasite, we conducted a genome-wide bioinformatics analysis of encoding proteins to functionally classify and characterize all the TM proteins in *E. histolytica*. In the present study, 692 TM proteins have been identified, of which 546 are TM transporters. For the first time, we report a set of 141 uncharacterized proteins predicted as TM transporters. The percentage of TM proteins was found to be lower in comparison to the free-living eukaryotes, due to the extracellular nature and functional diversification of the TM proteins. The number of multi-pass proteins is larger than the single-pass proteins; though both have their own significance in parasitism, multi-pass proteins are more extensively required as these are involved in acquiring nutrition and for ion transport, while single-pass proteins are only required at the time of inciting infection. Overall, this intestinal parasite implements multiple mechanisms for establishing infection, obtaining nutrition, and adapting itself to the new host environment. A classification of the repertoire of TM transporters in the present study augments several hints on potential methods of targeting the parasite for therapeutic benefits.

## 1. Introduction

*Entamoeba histolytica* is an important human parasite. It is an enteric protozoan parasite that is responsible for amoebic dysentery and invasive extraintestinal amoebiasis. Developing countries show higher prevalence of amoebiasis, as there is an inadequate barrier between human feces and drinking water. It is responsible for around 100,000 deaths each year, being the second most common cause of deaths due to parasitic disease [1,2]. One of the most critical molecules involved in interface functioning are the transmembrane (TM) proteins that fully or partially span the membrane. A typical genome consists of around 25–30% of TM proteins [3,4]. Several TM proteins penetrate the membrane bilayer from the outside to the inside of the cell. TM proteins perform numerous integral functions in the cells such as adhesion, signaling, transport of membrane-impermeable molecules, cell recognition, cell–cell communication, etc. Communication is one of the most important functions of the TM protein, which helps in signaling the cell regarding the external environment. Transmembrane proteins also help in regulating the interchange of molecules across the membrane; thus, functioning as regulators of the cell. In the case of parasites, these also play an important role as transporters/channels of nutrients from the host. TM proteins can be divided into three domains: (i) the domain in the bilayer, (ii) the extracellular domain, and (iii) the intercellular domain [5]. They mediate the shuttling of metabolites that are vital for its survival across membranes in the organelles of the parasite. Many of these TM proteins are ideal drug targets, as these are absent in the host [6]. Transmembrane proteins are an important means of interaction in the host–parasite relationship. In the case of *E. histolytica* infections, the interactions made by TM proteins is suggested to be of prime importance for adhesion, tissue invasion, and initiation of colitis and liver abscess formation. Seeing the key roles of membrane proteins, it is necessary to understand membrane proteins as these represent more than 60% of drug targets [7,8]. In many respects, it is easier to investigate membrane proteins computationally than experimentally, due to the uniformity of their structures and interactions [9,10]. Considering the biological and pharmacological significance of TM proteins, comprehensive analysis will help in understanding the structure–function relationships of these TM proteins in a host–parasite interaction and will direct research towards the development of better therapeutic controls. So far, no such effort to look into TM proteins/channels/transporters in *E. histolytica* has taken place. To identify the complete set of TM transport proteins that are thought to be of prime importance for host–parasite interactions, the complete genome of *E. histolytica* was explored, implementing bioinformatics analysis. We identified 692 TM proteins, which constitute 16.1% of the *E. histolytica* genome, which is much less compared to other organisms.

## 2. Materials and Methods

**Figure 1:**
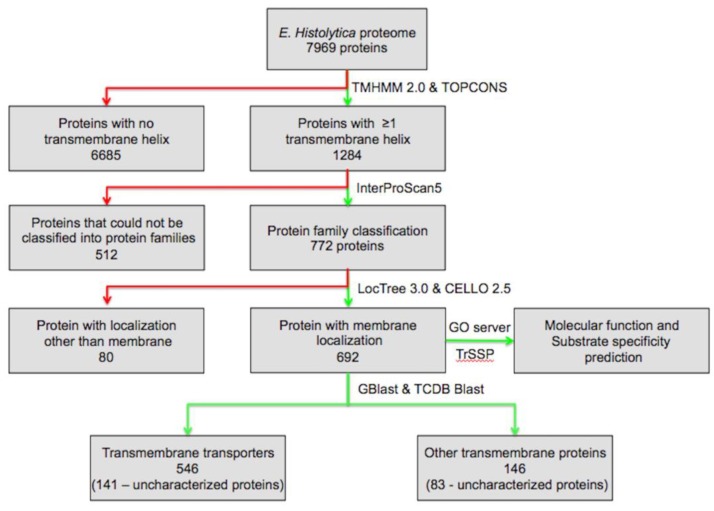
Flow scheme for the identification and classification of transmembrane (TM) proteins and TM transporters in *Entamoeba histolytica*. Green lines represent subsets of proteins that are included for further analysis, while red lines represent protein subsets that are excluded from further analysis.

To identify the comprehensive set of TM proteins, the predicted protein sequences from the open reading frames (ORFs) of the *E. histolytica* genome were retrieved from UniProt release 2014_01 [11]. To identify the TM proteins, we looked for proteins with TM helices, using TMHMM 2.0 and TOPCON servers [4,12] across the *E. histolytica* proteome, consisting of 7959 proteins. According to TMHMM and TOPCON results, proteins with one or more predicted helices were retained, while proteins with no helix were discarded, which resulted in 1284 proteins with one or more TM helix. Functional classification of the proteins was carried out using a standalone version of InterProScan5 version 5.27–66.0 (http://www.ebi.ac.uk/interpro/interproscan.html) by querying each of the TM helix containing proteins against the InterPro database [13], which resulted in the identification of 772 proteins belonging to various protein families, while for rest of the proteins no hits were obtained. The subcellular localization of the proteins was checked using standalone version of LocTree 3.0 and CELLO v.2.5 server [14,15]; proteins predicted to be localized at the membrane by either LocTree 3.0 or CELLO v.2.5 were considered. Overall, 692 proteins were found to be associated with the membrane. Identification of the signal peptides was done at the SignalP server [16,17]. Classification of the TM proteins into different classes of transporters was done on the basis of the Transport Classification Database (TCDB) [18,19,20]. A set of 692 proteins was screened against the TCDB for transport protein homologues by using the standalone program GBlast [21] and TCDB Blast [18], which resulted in the classification of 546 proteins with e-value cut-off scores of ≤0.01. For the proteins where the results of the protein family classification and TCDB classification were not similar, these proteins were analyzed individually using TCDB Blast, and these hits were grouped into unclassified TM proteins. The substrate specificity of each of the protein was predicted using the Transporter Substrate Specificity Prediction Server-TrSSP [22]. Molecular functions of the proteins were analyzed at Gene Ontology server [23,24] (Figure 1).

## 3. Results

### 3.1. Identification and Functional Characterization of Transmembrane Proteins and Transmembrane Transporters

Transmembrane proteins span the plasma membrane through α-helical structures, thus, the TMHMM and TOPCONS analysis of the *E. histolytica* proteome consisting of 7969 proteins resulted in the identification of 1284 proteins with one or more TM helices, i.e., 16.1% of the proteome. Functional prediction of the 1284 proteins through InterPro resulted in an identification of function for only 772 proteins. Since we were only interested in TM proteins, the localization of all of the 772 proteins was checked through LocTree 3.0 and the CELLO v.2.5 server, which resulted in the identification of 692 proteins that were located at the membrane. Further molecular function and substrate specificity of the subset of 692 proteins was analyzed, along with transporter classification. An E-value cut-off of 0.01 was used for the Gblast results, and the proteins for which the hits were not matching were analyzed manually using TCDB Blast. Gblast and TCDB analysis resulted in the identification and classification of 546 TM transport proteins. In the present work, a larger repertoire of TM proteins and TM transporters were identified than in earlier reports [25,26,27]. TransportDB, a database of a complete set of transport proteins in sequenced genomes, reports only 191 transporting proteins in *E. histolytica*, including 102 secondary transporters, 81 adenosine triphosphate (ATP)-dependent transporters, seven ion channels, and one unclassified protein [25,26], while in another study, 174 proteins that act as various types of transporters were reported, including 64 ATP-dependent transporters, seven ion channels, 103 secondary transporters, and one unclassified protein [27]. Thus, we present a comprehensive set of 692 TM proteins, out of which 546 are TM transport proteins, with their functional characterization (Table 1).

**Figure 2:**
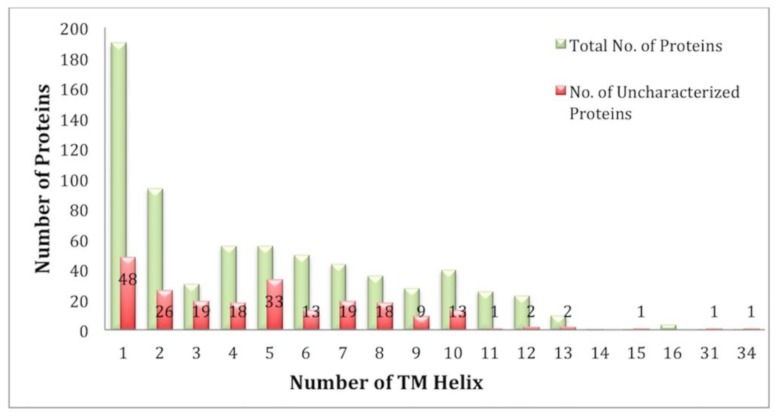
Overviews of the number of TM proteins having single or multiple TM helices. Red bars represent the share of uncharacterized proteins.

Analysis of the TM segments revealed that the number of TM helixes in the *E. histolytica* genome varied from 1 to 16, and there were two proteins with 31 and 34 TM helixes. The number of single-pass proteins was 190, while there were 502 multi-pass TM proteins (Figure 2).

### 3.2. Classification of Transmembrane Transporters

According to the transport protein classification (TC) system transport proteins are classified into five well defined (classes TC 1. to TC 5.) and two poorly defined classes (classes TC 8. and TC 9.), while classes 6 and 7 have not been assigned in this classification system [17,18,19]. The well-defined categories are: channels (TC 1.), secondary carriers (TC 2.), primary active transporters (TC 3.), group translocators (TC 4.), and TM electron carriers (TC 5.), while the other two classes that are not well defined according to TC system include: Accessory factors that are involved in transport (TC 8.), and incompletely characterized transport systems (TC 9.). In the present study, we observed TM transporters from all the TC classes in *E. histolytica* (Table 2).

#### 3.2.1. Channels (TC 1.)

Channels or pores enable the flux of solutes or ions across the membrane, which move simultaneously to both the extracellular and intracellular regions, triggering the environment-driven responses. In the present study, we have identified 11.6% (80) of TM proteins that function as various types of channels. This class of TM transporters have almost a similar quantity of singlepass (39.7%) as well as multi-pass (60.3%) proteins (Figure S1). These channel proteins in *E. histolytica* were distributed across six subclasses (Table 2).

##### α-Type Channels (TC 1.A.)

These types of transporters are recognized by TM *α*-helical secondary structure building sequences. α-type channels constituted about 56.3% of all the channel proteins. Several families of α-type channel proteins were identified, and some of the important ones included three proteins belonging to the family of voltage-gated ion channels (T.C. 1.A.1), which are ion-selective channel proteins. A single protein from the family of transient receptor potential Ca^2+^ channels (T.C. 1.A.4) i.e., a family of cation channels, was seen. Two members from a large and diverse family i.e., the major intrinsic proteins (T.C. 1.A.8) were present; this family includes proteins that function in transporting solutes including water, small neutral carbohydrates, urea, NH_3_, CO_2_, H_2_O_2_, and ion transport by an energy-independent mechanism [28,29]. There was one protein from (T.C. 1.A.16), the formate-nitrite transporter (FNT) family. Formate-nitrite transporter family members are prokaryotic proteins that transport structurally related compounds such as formate, nitrite, and hydrosulfide. There was one member from the family of small conductance mechanosensitive ion channels (T.C. 1.A.23); these proteins function as electromechanical switches and have the ability to sense the physical state of lipid bilayers. There were three proteins from the family of CorA metal ion transporters (T.C. 1.A.35), which function as metal ion transporters. Proteins from the Golgi pH regulator family (T.C. 1.A.38), which has only one member at TM region in *E. histolytica*, regulate the acidic pH i.e., for trafficking, processing, and glycosylation of proteins and lipids involved in transport [30]. Proteins from this family are novel anion channels that are crucial for acidification and functions of the Golgi apparatus [30]. There were four proteins that are important in calcium-mediated lysosomal fusion [31,32], from the family of presenilin endoplasmic reticulum (ER) Ca^2+^ leak channels (presenilin) (T.C. 1.A.54). Three members from the family of mechanical nociceptors, piezo (T.C. 1.A.75) were found in *E. histolytica*, which are multi-pass, mechanically activated cation channels [33]. We observed two homologues from the family of magnesium transporter 1 (MagT1) members (T.C. 1.A.76); MagT1 family members are generally involved in Mg^2+^ transport, and they consist of 335 amino acids and possesses five TM segments [34]. Magnesium transporter 1 family proteins identified in *E. histolytica* are less than 150 amino acids and have only three TM segments. There are six proteins from the family of Mg^2+^/Ca^2+^ uniporters (MCU) (T.C. 1.A.77). Sixteen homologues of the family of mechanosensitive channels (T.C. 1.A.87) were seen in *E. histolytica*, and they function in Ca^2+^ import and export [35].

##### Pore-Forming Toxins (Proteins and Peptides) (TC 1.C.)

A large number of important pathogens utilize pore-forming toxins for virulence. Transmembrane pores are formed in the membrane of target cell by the proteins that are synthesized by another cell and secreted for insertion [36]. There were 13 members identified in this class which belong to six distinct families, including one protein from the *Serratia*-type pore-forming toxin (S-PFT) family (TC 1.C.75), one protein from the *Botulinum* and tetanus toxin family (*TC 1.C.8*), two proteins from the clostridial cytotoxin (CCT) family (TC 1.C.57), one protein from the SphH hemolysin (SphH) family (TC 1.C.67), and one protein from the hemolysin III family (TC 1.C.113), while most of the proteins (eight) were from the *Bacillus thuringiensis* vegetative insecticidal protein-3 family (TC 1.C.105).

##### Vesicle Fusion Pores (TC 1.F.)

Multiple members (14) from the subclass vesicle fusion pores were identified; all of these were from the family of synaptosomal vesicle fusion pores (TC 1.F.1). These proteins function as power engines to bring the membranes together [37].

##### Membrane-Bound Channels (TC 1.I.)

A single member from this subclass was seen belonging to the family of eukaryotic nuclear pore complexes (TC 1.I.1.). Proteins from this family facilitate molecular trafficking between the nucleus and the cytoplasm, and they are an integral feature of the eukaryotic cell [38].

##### Cell Fusion Pores (TC 1.N.)

This subclass of channels includes proteins that are involved in pore formation in the membrane [18]. There were three members in *E. histolytica* belonging to the endoplasmatic reticulum fusion GTPase, atlastin (atlastin) family (TC 1.N.5).

##### Non-Enveloped Virus Penetration Complex (TC 1.P.)

In *E. histolytica* there were four proteins from this class of transporters, which has only one family, the polyoma virus SV40 ER penetration channels (TC 1.P.1). It has been demonstrated that the proteins from this family reach the cytosol by penetrating the endoplasmic reticulum membrane to cause infection [39].

#### 3.2.2. Secondary Carriers (TC 2.)

This class of transporters utilizes a carrier-mediated mechanism to catalyze the transport. Secondary carriers rather than primary active transporters dominate TM transport in eukaryotes. In *E. histolytica 134* (19.4%) of secondary carriers were found. Secondary carriers from only three subclass TC 2.A., TC 2.C., and TC 2.D. were seen in this parasite (Table 2). The percentage of secondary transporters in different eukaryotes ranges between 44% and 85% [40], but in *E. histolytica*, it was observed that the percentage of secondary carriers was lesser than the other eukaryotes. Among the secondary carriers, 92.5% of proteins are multi-pass, while 7.5% was single-pass transporters (Figure S1).

##### Porters (Uniporters, Symporters, Antiporters) (TC 2.A.)

A total number of 131 secondary carriers were from this subclass. This subclass was divided into 129 families, and in case of *E. histolytica*, proteins from 29 different families of porters were present (Table S1). Some of the important families represented in this subclass included the major facilitator superfamily (TC 2.A.1) i.e., a diverse superfamily catalyzing uniports, symports, and antiports, 29 proteins were seen from this family. The drug/metabolite transporter superfamily (TC 2.A.7) consisted of 19 proteins in *E. histolytica.* The members of this superfamily are mostly nucleotide–sugar transporters functioning in the endoplasmic reticulum and Golgi of eukaryotic cells [41]. The amino acid/auxin permease family (TC 2.A.18) is represented by 14 members, functioning in transporting single/multiple amino acids.

##### Ion-Gradient-Driven Energizers (TC 2.C.)

The members of this subclass catalyze the passive transport of solutes across the membrane. *E. histolytica* encodes one protein from this subclass that belongs to its only family, TonB-ExbB-ExbD/TolA-TolQ-TolR outer membrane receptor energizers and stabilizers (TC 2.C.1). The TolA system transports group A colicins, and reports suggest that the loss of one of its proteins leads to increased sensitivity to drugs and bile salts [42].

##### Transcompartment Lipid Carriers (TC 2.D.)

As the name suggests the members of this subclass are involved in transporting lipids across compartments, which helps to maintain membrane lipid homeostasis. There are two members represented in the family (TC 2.D.1).

#### 3.2.3. Primary Active Transporters (TC 3.)

The active transport of solutes against a concentration gradient is derived by a primary source of energy such as chemical, electrical, or solar energy [18]. Primary active transporters constituted about 12.4% (86) of the total TM proteins in *E. histolytica*. In different eukaryotes, the percentage of primary active transporters ranges between 11% and 22% [40]. Thus, it is evident that *E. histolytica* also has a similar repertoire of primary active transporters, like other eukaryotes. Primary active transporters are divided into five subclasses, but in the present study, primary active transporters from only two subclasses, 3.A and 3.D., were observed (Table 2). The percentage of single-pass proteins in this class was 19.3%, and for multi-pass proteins it was 80.7% (Figure S1).

##### P–P-Bond-Hydrolysis-Driven Transporters (TC 3.A.)

The proteins of this subclass function in the active uptake or elimination of solutes by hydrolyzing the diphosphate bond of either inorganic pyrophosphate, ATP, or another nucleoside triphosphate [18]. In *E. histolytica*, 95.3% of primary active transporters belonged to this subclass. Some of the major families included the ATP-binding cassette (ABC) Superfamily (TC 3.A.1), which consists of 27 members. This superfamily includes both uptake and efflux transport systems where ATP hydrolysis is not energized by protein phosphorylation [43]. *E. histolytica* encodes five proteins from the superfamily of H^+^- or Na^+^-translocating F-type, V-type, and A-type ATPases (TC 3.A.2). The proteins from this superfamily are involved in energy interconversion in the mitochondria. The P-type ATPase superfamily (TC 3.A.3) catalyzes the efflux of mono-, di-, and tri-valent cations, and we have seen 19 members from this superfamily in *E. histolytica.* Of several protein secretion systems (14), components were also identified from the family TC 3.A.16.

##### Oxidoreduction-Driven Transporters (TC 3.D.)

This subclass of transporters includes proteins that carry out transport from a reduced to an oxidized substrate, and the transport system is energized by the exothermic flow of electrons [18]. In *E. histolytica*, 4.7% of the primary active transporters constituted this subclass, which further belonged to two different families including three members from the proton-translocating transhydrogenase family (TC 3.D.2) that catalyze the TM translocation of one proton per hydride transferred between NADH and NADP, and one member from the superfamily of proton-translocating quinol:cytochrome C reductases (TC 3.D.3) which functions in transferring electrons from a quinol to cytochrome c and links electron transfer to proton translocation [44].

#### 3.2.4. Group Translocators (TC 4.)

Group translocators modify the transported substrate during the transport process via combined chemical and vectorial reactions. The TM transporters belonging to this class were 1.7% (12) (Table 2). All of the members of this class are multi-pass transporters. All of the group translocators were observed from the two subclasses i.e., three proteins from the polysaccharide synthase/exporters (TC 4.D.) and nine proteins from the choline/ethanolamine phosphotransferase 1 (*TC 4.F.*) subclass.

#### 3.2.5. Transmembrane Electron Carriers (TC 5.)

Members of this class catalyze electron flow across biological membranes, where donors are located at one side and acceptors at the other side of the membrane [18]. We identified two proteins from this class belonging to the family of gp91^phox^ phagocyte NADPH oxidase-associated cytochrome b558 (TC 5.B.1). Both of the proteins from this class are multi-pass transporters, having 5–7 TM helix.

#### 3.2.6. Accessory Factors Involved in Transport (TC 8.)

This class of transporters consists of proteins that are not involved in transport directly, but they couple to known transporters to carry out the process. The percentage of TM transporters from this class was 9.7% (67) (Table 2). All of these proteins were from the subclass 8.A., which consists of auxiliary transport proteins. Most of these proteins (60.6%) were multi-pass proteins (Figure S1).

##### Auxiliary Transport Proteins (TC 8.A.)

All of these proteins were from the subclass of auxiliary transport proteins (TC 8.A.), belonging to 12 different families, of which one of the mostly dominated family was the basigin (basigin) family (*TC 8.A.23*), with 39 members. Proteins from this family are involved in regulating transporters [45].

#### 3.2.7. Incompletely Characterized Transport Systems (TC 9.)

In this class of transporters all the protein families from unidentified classification are grouped. The repertoire of incompletely characterized transport systems consisted of 24.0% (166) of TM transporters (Table 2). These proteins were from the two main subclasses i.e., recognized transporters of unknown biochemical mechanisms (TC 9.A.) and putative transport proteins (TC 9.B.). Likewise, other classes this class is also dominated by multi-pass proteins (72.3%) (Figure S1).

##### Recognized Transporters of Unknown Biochemical Mechanism (TC 9.A.)

The members of this subclass are recognized as transporters, but the mode and mechanism of action is unknown. In *E. histolytica* this subclass constitutes 28.9% of its class (TC 9.). Transporters from ten different families were identified in this subclass. The largest family was the autophagy-related phagophore-formation transporters (TC 9.A.15); eukaryotes employ autophagy to degrade damaged organelles and proteins [46].

##### Putative Transport Proteins (TC 9.B.)

This subclass includes putative uncharacterized transport proteins. It constitutes 71.1% of its class (TC 9.). Transporters from 33 families of this subclass were identified in *E. histolytica.* Some of the important families include putative heme handling protein (TC 9.B.14), consisting of 15 members; the exact function of this family of transporters is not known, but they are suggested to be involved in heme transport [47]. Another family consisting of 23 members was the Huntington-interacting protein 14 (HIP14) family (TC 9.B.37); the members of this family are reported to be divalent cation transporters [48]. A total of 12 proteins were identified from the family of lipoprotein signal peptidase/phosphatase/lead resistance fusion proteins (TC 9.B.105); transporters from this family are suggested to be potential Pb^2+^ exporters. The integral membrane glycosyltransferase family 39 (TC 9.B.142) functions in glycosyl transfer and sometimes transfers its own substrate across the membrane. *E. histolytica* constituted 15 members from this family.

### 3.3. Uncharacterized Proteins

In the present study, 224 uncharacterized proteins were identified as TM proteins (Table S1). An analysis of all the uncharacterized TM proteins revealed that 190 of these were single-pass TM proteins, while rest (34) were multi-pass proteins. A subset of uncharacterized TM proteins (141) was classified as TM transporters for the first time in the present study. Further, functional analysis and classification revealed that 33.8% (27) of the class channels constituted uncharacterized proteins, and similarly, 26.8% (36) of secondary carriers, 16.3% (14) of primary active transporters, 100% (2) of TM electron carriers, 25.7% (17) of accessory factors involved in transport, and 30.8% (45) of incompletely characterized transport systems were uncharacterized proteins (Table 2).

### 3.4. Substrate Specificity

The prediction of substrate specificity resulted in the identification of the substrate for 43% of the candidate transport proteins identified in the *E. histolytica* genome. These proteins showed adequate homology to proteins of known substrate specificities (Table S1).

## 4. Discussion

### 4.1. Identification and Functional Characterization of Transmembrane Transport Proteins

Parasitic protozoa depend on scavenging nutrients from their hosts, due to the loss of key biosynthetic pathways. Transport proteins are critical for their survival, as these ensure the nutritional requirements of the parasite. The availability of nutrients is made possible by plasma membrane transport proteins, which can be potential therapeutic targets. Thus, identifying the repertoire of TM proteins and understanding their function is important. In the present study, we have reported 16.1% TM proteins in *E. histolytica*, while an earlier study reports 693 surface proteins, of which 3.2% are membrane proteins that function in adhesion or act as transporters [49]. In general, TM proteins constitute 25–30% of the genome, but in protozoans, they are reduced in comparison to the free-living organisms. This may be due to extracellular parasites only needing TM transporters when they are inside the host; earlier studies support that increased functional diversification of the parasite transporter proteins is responsible for the reduction of these transporters [27]. Single-pass membrane proteins exhibit tremendous functions on their extracellular sides, facilitating parasites to invade and propel inside host plasma membranes [50]. Protozoans extensively employ multiple pass TM proteins for nutrient and ion transports. In general, it is evident that protozoan parasites rely greatly on multiple pass proteins rather than single-pass proteins for their infectivity and survival [51]. The set of identified 546 TM transporters includes some previously reported TM transporters, indicating the accuracy of the identification pipeline. These transport proteins include P-glycoprotein-5 transporters—EHI_175450, EHI_125030, EHI_075410, ABC transporters—EHI_095820, EHI_178050, EHI_178580, and a major facilitator family member EHI_173950 [52].

### 4.2. Classification of Transmembrane Transporters

#### 4.2.1. Channels

Channels and pores are modulated by electrochemical potential for delivering essential nutrients and disposing cellular waste [53]. Proteins from this class play an essential role in regulating ion concentration, and they are vital for parasite survival. α-type channels typically employ energy-independent processes to catalyze the movement of solutes through an aqueous pore or channel, suggesting that these putative channels have widespread and conserved physiological functions. Many drugs that obstruct K^+^ channel (an α-type channel) activity are known to be toxic to the parasite, as these drugs alter cellular ion homeostasis, leading to the decreased survival of parasites [54]. Similarly, mechanosensitive channels play crucial roles in cell physiology, and the dysfunction of mechanosensitive channels has great effects on the functions of the cell [55]. Ion channels are also vital for maintaining the membrane potential, osmotic balance, and driving transport processes across the membrane. These channels were involved in transporting ions such as sodium, calcium, potassium, chloride, etc. It has been seen that the processes that are dependent on ion channels contribute to the excystation and metacystic development of the parasite inside the host, thus playing a critical role in pathogenesis [56]. The pore-forming toxin proteins help in pore formation and the penetration of the parasite in the epithelial cell membrane of the host intestine [57], and they are insecticidal in nature [36]. These results imply a crucial role played by the different type of channels in parasite biology, suggesting that developing drugs for these parasite-specific channels may be a novel approach for a therapeutic strategy.

#### 4.2.2. Secondary Carriers

Several lines of evidence suggest that representatives from secondary transporters are crucial for the pathogenesis and survival of the parasite. In *E. histolytica*, secondary carriers constitute the largest class, having transporters from several TC families. It was demonstrated that the plasma membranes of *Leishmania donovani*, a protozoan parasite, were enriched in acid phosphatase activity, indicating the integral role of phosphatases in parasitism [58]. In our previous study, it was suggested that several phosphatases are involved in cation TM transport activity and ion channel activity [59,60,61]. Cell signaling has been found to regulate lipid phosphate phosphatases (LPPs) by adjusting the concentrations of lipid phosphates against their dephosphorylated products [62]. Phosphoesterases play an integral role in cyclic nucleotide signaling. Several nucleotide sugar transporters were identified that function in transporting multiple nucleotide sugars [41]. Targeting these will greatly facilitate the development of parasite-specific drugs [63]. Proteolysis is largely conserved throughout different life forms, and TM proteases from all four major proteolytic classes are utilized by protozoan parasites and have been seen to be involved in important functions, including invasion, the catabolism of host proteins for nutritional purposes, the degradation of immune response mediators, and sometimes some unexpected functions. [64,65]. The rhomboid protease of *E. histolytica* has been demonstrated to play an important role in the attachment to and the phagocytosis of host cells [66], and cysteine proteases have the potential to contribute to host tissue breakdown in vivo [67]. These results suggest that the parasite is unable to meet its nutrition and survival requirements through primary active transporters; thus, it heavily depends on secondary transporters that utilizes the energy stored in the concentration gradient that is established by the primary active transporters.

#### 4.2.3. Primary Active Transporters

In *E. histolytica*, the percentage of primary active transporters is much lower than the secondary transporters; this suggests that *E. histolytica* depends primarily on other sources of energy rather than energy that is obtained from ATP hydrolysis. Since the primary active transporters obtained energy from ATP hydrolysis, these membrane proteins transport many chemotherapeutic drugs away from their targets inside the parasites [68]. Several lines of evidence suggest the role of ATP-dependent transporters in drug resistance [69]. Transmembrane kinases that are also ATP dependent, upon invasion, help to overcome a variety of challenges that need the capacity to obtain sufficient nutrients from the host, perform chemotaxis, adhere, kill, and ingest human cells [70,71]. It has been suggested that the large family of TM kinases in *E. histolytica* has an important role in the amebic response to the environment and immune system evasion [72]. The host tissue suffers substantial destruction and inflammation upon the invasion of the parasite into the colon; on the other hand, the parasite encounters reactive oxygen and nitrogen species which causes large scale variations in gene expression profiles, which helps the parasite in adapting to the host environment. Host cell invasion is an important aspect of *E. histolytica* parasitism, it has been demonstrated that a membrane-bound efflux pump p-glycoprotein belonging to the ABC transporter superfamily is associated with drug resistance in protozoan parasites. The inhibition of the p-glycoprotein in *T. gondii* resulted in the failure of parasite invasion, while its overexpression is associated with multi-drug resistance [73]. Phospholipid-transporting P-type ATPases are reported to have an important role in parasite virulence, and their overexpression enhances parasite survival under oxidative stress [74]. ATP4, a P-type ATPase of *Plasmodium falciparum*, is involved in sodium export coupled with proton import, which is lethal to the parasite upon blockade, and cell death may occur due to acidification of the cytosol, osmotic swelling, breakdown of the electrochemical potential, or a combination of these, but the exact reason is unknown [75,76,77,78]. Oxidoreduction-driven transporters carry out the transport of solutes from a reduced to an oxidized substrate by utilizing the energy derived from an exothermic flow of electrons. In *Xylella fastidiosa*, the oxidoreduction-driven transport systems present a complete respiratory string that enables ATP synthesis through an electrochemical proton motive force [79]. Parasites’ unique oxidoreduction-driven transporters are the choke points of metabolism and pharmacological targets.

#### 4.2.4. Group Translocators

In *E. histolytica*, group translocators from only one subclass, a putative acyl-CoA transporter, was predicted. This subclass is suggested to be critical for latent infection, as it helps in adaptation to the lipid-rich, oxygen-poor granulomas [80]. A glycosyltransferase family protein found in *E. histolytica*, synthesizes carbohydrate moieties of the glycoproteins and glycolipids and plays a very important role in several cellular functions [81]. The membrane-bound acetyltransferases play a part in nutrient acquisition in African trypanosomes [82].

#### 4.2.5. Transmembrane Electron Carriers

This class includes the proteins that catalyze electron flow across biological membranes. The subclass *TC 5.B.*, includes TM single-electron carriers. These proteins are essential for cellular energetics. The scarcity of TM electron carriers can be explained by that fact that several classical cell organelles like peroxisomes, well-defined Golgi apparatuses, and a rough endoplasmic reticulum are missing due to the early evolutionary diversification of *E. histolytica* [83], thus reducing the requirements of TM electron carriers that act across biological membranes.

#### 4.2.6. Accessory Factors Involved in Transport

These proteins facilitate the transport of ions/solutes across one or more biological membranes, but they themselves do not participate directly in transport. Several membrane fusion proteins were identified that are not involved in transport but facilitate transport that is driven by the energy source of cytoplasmic membrane transporters [18].

#### 4.2.7. Incompletely Characterized Transport Systems

A large number of TM transporters were observed from the two subclasses of incompletely characterized transport systems. The first subclass (*T.C. 9.A.*) consists of known transporters, while the other dominant subclasses (*T.C. 9.B.*) consists of unclassified transporters. Since a huge percentage of these proteins are uncharacterized transporters, an analysis of their functions and modes of transport will be beneficial for therapeutic purposes.

#### 4.2.8. Uncharacterized Proteins

A large number of uncharacterized proteins (224) were identified as TM proteins in the present study. Many of these uncharacterized proteins were classified into different classes of transporters. Earlier studies on transport proteins report only one uncharacterized protein as a TM transporter (UniProt ID C4LUW6), which was predicted to be from the putative arginine transporter family (*TC 9.A.5*) [25,26,27], but in the present study, this protein was not identified as there was no TM segment present in this protein. The importance of functional annotation of uncharacterized proteins can be ascertained by an earlier report where eight uncharacterized proteins from *Escherichia coli* were identified as outer membrane proteins through a bioinformatics approach, and the localization of five of them was confirmed experimentally, with one being found to be an outer membrane autotransporter [84]. The uncharacterized proteins identified in our study are predicted to be from diverse proteins families, and they are involved in important biological processes such as vesicular trafficking, protein folding, membrane transport, lipid metabolic processes, fatty acid synthesis, etc. (Table S1), indicating that these TM proteins are vital for the existence of the parasite; thus, further understanding of these will unknot the role of these proteins in host–parasite interaction in *E. histolytica*.

#### 4.2.9. Unclassified Transmembrane Proteins

In the present analysis we have identified 546 TM transport proteins, but there were several proteins that were recognized as TM proteins but could not be classified into a TC class. Protein family classification revealed that these proteins are involved in important functions like endoplasmic reticulum vesicle transporter, glycosyl transferases, major facilitator superfamily protein, rab-GTPase, serine/threonine phosphatases, serine/threonine kinases, etc. Many of these were uncharacterized proteins, and since we already performed a protein family classification, further investigations of their role in adhesion/transportation will offer novel hits for targeting the parasite.

## 5. Conclusions

Scavenging molecules from the host environment is still less understood in *E. histolytica* parasitism. We have observed a large set of TM proteins, a subset of which were identified as TM transporters. The TM protein repertoire of *E. histolytica* is smaller compared to other eukaryotes, which is justified by the functional diversification of the TM proteins in the parasite; also, *E. histolytica* regularly performs a high rate of phagocytosis and pinocytosis to obtain nutrients and to kill/eat host cells. The overrepresentation of multi-pass proteins may be due to the fact that the parasite requires more proteins for accomplishing its nutritional requirements than for adhering and infection. A classification of the TM transporters revealed that *E. histolytica* is affiliated with all of the main classes of transporters i.e., channels, secondary carriers, primary active transporters, group transporters, and transport electron carriers. Secondary carriers dominated over all the other main classes of transporters, followed by primary active transports, implying that the secondary carriers utilized the energy gradient established by ATP-driven hydrolysis (primary transporters) to scavenge molecules from the host, fulfilling its nutritional demand. A larger number of secondary carriers is also justified by the fact that, in the cyst stage, there is no nutritional and survival requirement by the parasite, but upon excystation, the parasite exploits its host for cell killing, inciting infection, and obtaining nutrition and reproduction in the new host environment, all by the energy-independent transport of solutes from its environment i.e., aided by secondary transporters. A vast number of uncharacterized proteins (224) were identified and functionally characterized as TM proteins; 63% of uncharacterized proteins were assigned to different transporter classes in the present study for the first time. The analysis reveals that these TM transport proteins play an important role in host–parasite interaction, and these are crucial for the survival of the pathogen. Moreover, several of these crucial transporters do not have homologues in higher eukaryotes. Therefore, the TM repertoire of *E. histolytica* represents an array of excellent potential therapeutic targets.

## Figures and Tables

**Table 1:** Comparison of TM transport proteins reported in previous studies with those that are identified in the present study.

	TransportDB [25,26]	Transport Proteins of Parasitic Protists [27]	Present Study
Total	191	174	546 (224)
Channel	7	7	80 (27)
Secondary carriers	102	103	134 (36)
Primary active transporter	81	64	86 (14)
Group transporters	-	-	12 (0)
TM electron carriers	-	-	2 (2)
Accessory factors involved in transport	-	-	66 (17)
Incompletely characterized transport systems	1	1	166 (45)

The numbers in parenthesis indicate uncharacterized/hypothetical proteins.

**Table 2:** Distribution of the TM transporters based on the transporter classification database into class and subclass in *E. histolytica*.

TC Class	Class Description	No. of Proteins	uncharP *	TC Subclass	Subclass Description	No. of Proteins
1	Channel	80	27	1.A	α-Type channels	45
				1.C	Pore-forming toxins	13
				1.F	Vesicle Fusion Pore	14
				1.I	Membrane-bounded Channels	1
				1.N	Cell Fusion Pores	3
				1.P	Non-Envelop Virus Penitration Complex	4
2	Secondary carriers	134	36	2.A	Porters (uniporters, symporters, antiporters)	131
				2.C	Ion-gradient-driven energizers	1
				2.D	Transcompartment Lipid Carrier	2
3	Primary active transporter	86	14	3.A	P-P-bond-hydrolysis-driven transporters	82
				3.D	Oxidoreduction-driven transporters	4
4	Group Transporters	12	0	4.D	Polysaccharide Synthase/Exporters	3
				4.F	Choline/EthanolaminePhosphotransferase1	9
5	TM electron carriers	2	2	5.B	Transmembrane 1-electron transfer carriers	2
8	Accessory factors involved in transport	66	17	8.A	Auxiliary transport proteins	66
9	Incompletely characterized transport systems	166	45	9.A	Recognized transporters of unknown biochemical mechanism	48
				9.B	Putative transport proteins	118
	Unclassified	146	83			

Transporter classes 6 and 7 have not been assigned in the Transport Classification (TC) system, and they are therefore absent. * uncharP—Uncharacterized proteins identified as TM proteins.

## Supplementary Materials

The following are available online at http://www.mdpi.com/2073-4425/9/10/499/s1, Table S1: Overview of transmembrane transporters in *E. histolytica*—classification, characterization, and functional analysis; Figure S1: Distribution of single-pass and multi-pass proteins in different classes of transporters (TC class) in *E. histolytica.*
