# Supplementary material for: Bioinformatics Analysis and Functional Prediction of Transmembrane Proteins in Entamoeba histolytica
Source: Genes (Basel). 2018 Oct 16;9(10):499. doi: 10.3390/genes9100499 (PMC6209943; doi:10.3390/genes9100499)
Supplement: Supplementary file 1 [file genes-09-00499-s001.zip › Figure S1.pdf]

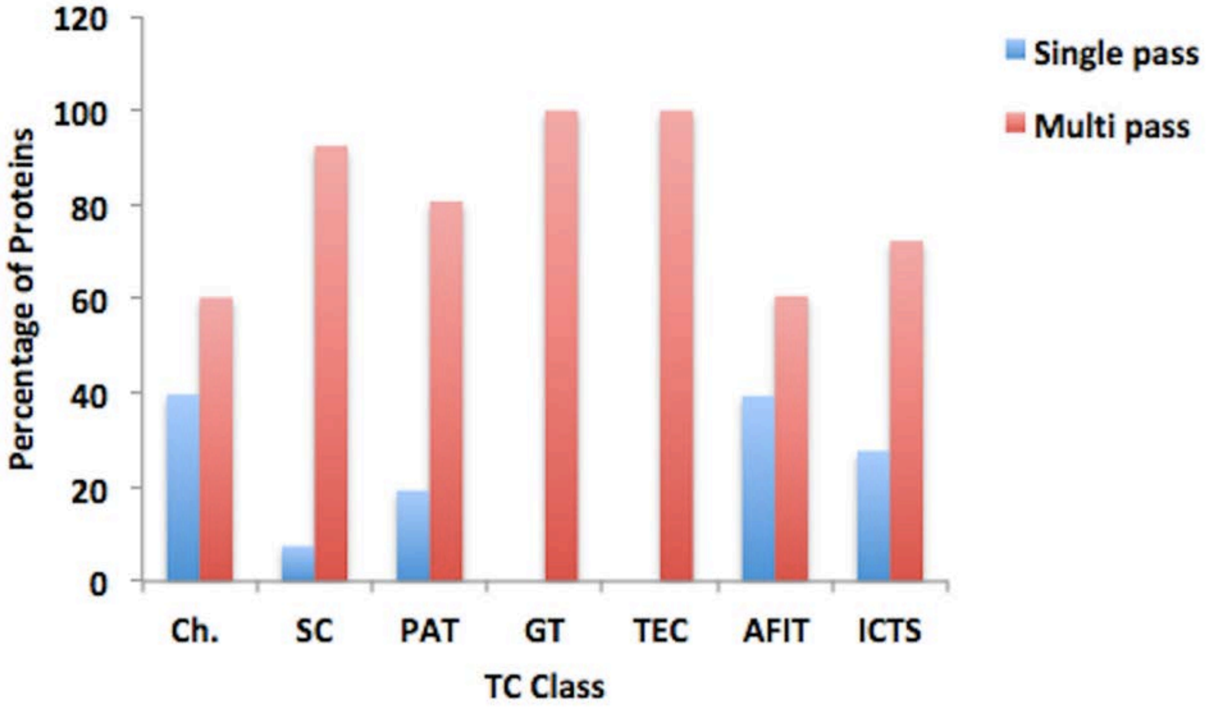


**Figure S1.** Distribution of single pass and multipass proteins in different classes of transporters in *E. histolytica*. (Ch.—channel, SC—secondary carriers, PAT—primary active transporters, GT—group translocators, TEC—Transmembrane electron carriers, AFIT—accessory factors involved in transport, ICTS—incompletely characterized transport systems).
